# Long Term Health-Related Quality of Life in Survivors of Sepsis in South West Wales: An Epidemiological Study

**DOI:** 10.1371/journal.pone.0116304

**Published:** 2014-12-30

**Authors:** Ceri E. Battle, Gareth Davies, Phillip A. Evans

**Affiliations:** NISCHR Haemostasis Biomedical Research Unit, Morriston Hospital, Swansea, United Kingdom; University of Leicester, United Kingdom

## Abstract

**Introduction:**

Survivors of sepsis report persistent problems that can last years after hospital discharge. The main aim of this study was to investigate long-term health-related quality of life in survivors of SIRS and sepsis compared with Welsh normative data, controlling for age, length of stay and pre-existing conditions. The second aim was to investigate any differences in long-term health-related quality of life specifically with the patients categorised into three groups; SIRS, uncomplicated sepsis and severe sepsis/septic shock.

**Methods:**

A prospective study design was used in order to investigate all sepsis patients either presenting to the Emergency Department or admitted to the Intensive Care Unit of a regional trauma centre. Baseline demographics, clinical characteristics and outcomes were collected and surviving patients were sent a SF-12v2 survey at between six months to two years post-hospital discharge.

**Results:**

Quality of life was significantly reduced in all patients when compared to local normative data (all p<0.0001). Reductions in the physical components of health-related quality of life were more pronounced in severe sepsis/septic shock patients when compared to uncomplicated sepsis and SIRS patients, when controlling for age, pre-existing conditions, hospital and ICU length of stay.

**Conclusions:**

This is the first observational study to specifically focus on the different groups of SIRS and sepsis patients to assess long-term quality of life. Local population norms were used for comparison, rather than UK-wide norms that fail to reflect the intricacies of a country’s population.

## Introduction

The incidence of sepsis has increased despite advances in supportive care and a number of national and international campaigns aimed at developing guidelines for the management of the condition. [1], [2] In the UK, severe sepsis is estimated to kill 37,000 patients annually, and consume 50% of critical care resources. [2] Severe sepsis is a common cause of Intensive Care Unit (ICU) admission and patients with sepsis are also at risk of other complications such as multi-organ failure and acute lung injury. [3] Pathophysiological mechanisms are not fully understood however severe infection initiates an inflammatory process that compromises the immune system, leading to more invasive infection, altered coagulation and cardiovascular instability and ultimately causing organ failure or death [3].

Survivors of sepsis report persistent problems that can last years after hospital discharge. These include physical, (weakness and dyspnoea) psychological (post-traumatic stress syndrome and depression), cognitive (poor concentration and memory loss) and social issues (delayed return to work and loss of earnings) [3]–[7].

Heyland et al (2000) reported that the long-term health related quality of life of survivors of sepsis was significantly lower than that of the general US population. [5] A more recent study reported that severe sepsis survivors have a significantly lower physical quality of life compared to population norms but mental quality of life scores were only slightly below population norms up to five years after hospital discharge. [4] Similar results were reported in another study, in which severe sepsis was independently associated with substantial and persistent new cognitive impairment and functional disability among survivors. [6] Hofhuis et al (2008) reported that physical and general health recovery is incomplete in survivors of severe sepsis at six months post-ICU discharge when compared with preadmission status [7].

Comparison between studies investigating long-term health-related quality of life outcomes in sepsis survivors is difficult. One of the main limitations described in the systematic review by Winters et al (2010) was that previous studies do not control for comorbidities when comparing to a control population. Another limitation they described was a lack of biomarkers for the sepsis patients investigated in the included studies. [3] They stated that because other conditions such as non-infectious systemic inflammatory response syndrome (SIRS) may masquerade as sepsis, they could not rule out the possibility of misclassification bias. [3] The main aim of this study therefore was to investigate long-term health-related quality of life in survivors of SIRS and sepsis compared with Welsh normative data, controlling for age, length of stay and pre-existing conditions. The second aim was to investigate any differences in long-term health-related quality of life specifically with the patients categorised into three groups; SIRS, uncomplicated sepsis and severe sepsis/septic shock.

## Materials and Methods

### Ethical Approval

Full ethical approval was given by the South West Wales Research Ethics Committee. Informed 2-stage written consent was given by patients with capacity to do so. Assent was obtained from personal or legal representation in cases where capacity to give informed consent was lacking.

### Setting and sample

A prospective study design was used in order to investigate all sepsis patients either presenting to the Emergency Department (ED) or admitted to the Intensive Care Unit (ICU) (from a hospital ward or peripheral hospital) of a large regional trauma centre in South West Wales from October 2011 to November 2013. Morriston hospital has approximately 90,000 presentations to the ED per year and serves a population of 450,000 people. A total of 106 patients were recruited and all patients were considered eligible as per the SIRS and sepsis criteria defined in 2003. [9] Using these criteria, SIRS was defined as a systemic inflammatory response that can be triggered by a variety of infectious and non-infectious conditions; uncomplicated sepsis is defined the presence of both infection and a systemic inflammatory response; severe sepsis was defined as sepsis complicated by organ dysfunction; and septic shock was defined as a state of acute circulatory failure characterized by persistent arterial hypotension unexplained by other causes.

Sepsis-related Organ Failure Assessment (SOFA) score was determined over the first 24 hours to assess organ function. [10] Patients were assigned to groups as follows: 1) Sterile SIRS 2) Uncomplicated sepsis 3) Severe sepsis or septic shock as per the criteria defined in 2003. [9] Assignment into groups was blinded and performed by an experienced intensive care specialist independent of the study.

A sample size of n = 100 was proposed for this study; calculated using a previously published Minimally Clinically Important Difference of 8.1 points (SD 9.7) using the SF-12v2 to assess quality of life. [8] The sample size, based on a three sample comparison of means was calculated at 17 per group for the SF-12v2 at 80% power with a two-sided, α error level of 0.05. This takes into consideration a 30% loss to follow up (mortality, drop-outs and non-response to the survey).

### Study design and outcome measures

Baseline demographics, clinical characteristics and outcomes were collected during hospital stay. These included age, sex, SOFA scores, pre-existing conditions, primary admitting diagnosis, hospital and ICU length of stay and mortality. Pre-existing conditions were scored using a previously published electronic version of the Charlson Comorbidity Index. [11] Primary admitting diagnosis was categorised into groups; respiratory, neurologic, cardiovascular, endocrine, renal and gastro-intestinal. In terms of the assessment of quality of life, this study used a cross sectional design surveying the survivors of sepsis at a single point in time (there was no baseline measure of health-related quality of life). Surviving patients were sent a postal survey at between six months to two years post-hospital discharge. A second survey was sent out to non-responders after two months.

The Short Form-12 version 2 (SF-12v2) is a multipurpose survey with 12 questions, (all selected from the SF-36 Health Survey) which investigate mental and physical functioning and overall health-related quality of life. It has been developed to provide a shorter alternative to the SF-36. [12] The SF-12 is a valid and reliable measure that is weighted and summed to provide easily interpretable scales for physical and mental Health Composite Scores (PCS & MCS) in which scores range from 0 to 100 (100 = optimal) [12], [13].

This instrument contains eight multi-item dimensions (ie, physical functioning [PF], role limitation due to physical problems [RP], bodily pain [BP], general health [GH], vitality [VT], social functioning [SF], role limitation due to emotional problems [RE], and mental health [MH]). The physical health summary score (PCS) reflects PF, RP, BP, and GH. The mental health summary scale (MCS) reflects VT, SF, RE, and MH. Answers were transformed, weighed, subsequently scored and aggregated to summary measures according to predefined guidelines. [14] The US normative data normally used to complete this transformation and analysis were replaced with Welsh normative data produced in 2011 [15].

### Statistical analysis

All statistical analyses were performed using IBM SPSS Statistics version 20 software. A baseline comparison of demographics, clinical characteristics and outcomes was completed with continuous data expressed as medians (interquartile ranges) and categorical data expressed as absolute values (percentages). Scores for each for the eight domains on the SF-12v2 were expressed as means (SD) as previously recommended. [16] These means (SD) were then compared to the Welsh normative data and also to Mid and West Wales normative data (geographical location of Morriston hospital) using independent t tests.

Data normality was assessed using the Kolmogorov-Smirnov and Shapiro-Wilk tests using an alpha value of 0.05. For the subgroup analysis, differences between the three groups were compared using Analysis of Covariance, in order to control for the possible confounding variables age, co-morbidity and hospital and ICU length of stay. Bonferroni correction was used to assess multiple comparisons across groups. All p values were two tailed and p values <0.05 were considered statistically significant.

## Results

A total of 109 patients who met study inclusion criteria presented to the ED during the data collection period. A mortality rate of 34% was recorded in this study. Three patients were of no-fixed-abode therefore a total of 69 surveys were sent out for completion. A total of 50 surveys (response rate 72%) were received by the end of the data collection period. There was no significant difference in any of the variables analysed between the responders and non-responders. The patients’ demographic and clinical characteristics are highlighted in Table 1. The only significant difference between the three groups was hospital and ICU length of stay.

**Table 1:** Demographic and clinical characteristics.

Characteristic	All patients n = 50	Group 1 n = 19	Group 2 n = 16	Group 3 n = 15
Age, years	58 (30)	52 (33)	46.5 (30)	68 (17)
Male, %	23 (46)	6 (32)	8 (50)	9 (60)
Female, %	27 (54)	13 (68)	8 (50)	6 (40)
SOFA score	3 (4)	3 (3)	3 (3)	5 (9)
CharlsonComorbidity Index	3 (4)	4 (5)	2 (4)	4 (5)
ICU LOS, days	0 (4)	0 (1)	0 (5)	4 (21)^Δ^ ‡
Hospital LOS, days	7 (15)	6 (16)	5.5 (10)	19 (29)^Δ^ ‡
Diagnostic groups, %				
Respiratory	18 (36)	3 (16)	8 (50)	7 (47)
Gastrointestinal	9 (18)	4 (21)	1 (6)	4 (27)
Neurologic	1 (2)	1 (5)	0 (0)	0 (0)
Endocrine	10 (20)	9 (47)	0 (0)	1 (7)
Renal	8 (16)	2 (11)	5 (31)	1 (7)
Other	4 (8)	0 (0)	2 (13)	2 (13)

Δ denotes a significant difference between group 1 and 3. (p<0.05).

‡ denotes a significant difference between group 2 and 3. (p<0.05).

All results are presented as medians (IQR) or absolute values (%).

Table 2 illustrates the results of the SF-12v2 survey, comparing all the SIRS and sepsis patients (n = 50) with the Welsh population and Mid/West Wales population. Significant differences were recorded for each of the eight health domains, between the SIRS and sepsis patients and both populations. Means and standard deviations are presented for each domain.

**Table 2:** Short form-12 mean scores (SD) for survivors of SIRS/sepsis compared to Welsh population and Mid/West Wales population.

Domains	All sepsis patients	All Welsh Population	p value	Mid/West Wales Population	p value
Physical functioning	35.2 (11.3)	77.8 (30.0)	<0.0001	77.5 (30.8)	<0.0001
Role physical	37.8 (9.5)	78.3 (32.3)	<0.0001	78.1 (31.9)	<0.0001
Bodily pain	36.8 (12.7)	70.1 (28.9)	<0.0001	69.4 (28.9)	<0.0001
General health	34.8 (10.9)	66.2 (24.0)	<0.0001	66.2 (23.9)	<0.0001
Vitality	39.8 (10.0)	57.2 (22.3)	<0.0001	57.7 (22.6)	<0.0001
Social functioning	36.4 (12.0)	80.2 (28.1)	<0.0001	80.4 (27.9)	<0.0001
Role emotional	36.8 (12.8)	87.0 (26.0)	<0.0001	87.4 (25.4)	<0.0001
Mental health	44.8 (13.5)	74.0 (18.9)	<0.0001	74.7 (18.7)	<0.0001

All results are presented as means (SD).

**Figure 1:**
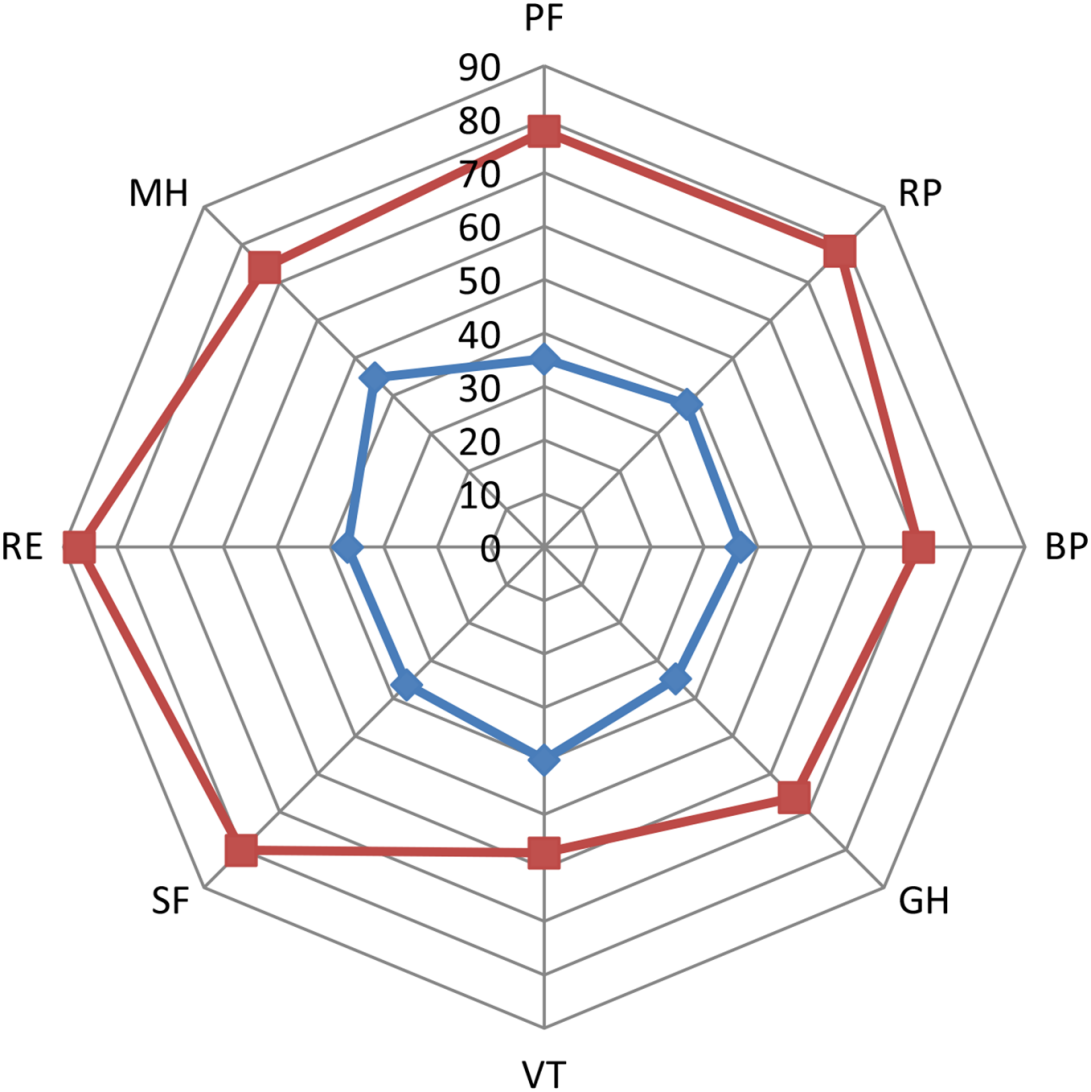
Health-Related Quality of Life in sepsis survivors at six months to two years compared to the Welsh Population. Radar chart of the health related quality of life in long term survivors of sepsis compared to Welsh population. Blue line with squares: all sepsis group results. Red line with diamonds: Welsh population results. PF: physical functioning, RP: role limitation physical, BP: bodily pain, GH: general health, VT: vitality, SF: social functioning, RE: role limitation emotional, MH: mental health.

Fig. 1 is a radar chart which illustrates the difference in each of the eight health domains between the sepsis patients and the Welsh population.

The ANCOVA results in Table 3 highlight the Short form-12 mean scores comparing the SIRS, uncomplicated sepsis and severe sepsis/septic shock patients, when controlling for the variables age, pre-existing conditions, hospital length of stay and ICU length of stay. Significant differences were found between groups 1 and 3 all the domains accept the mental health, role emotional and mental component scores. Significant differences were reported between groups 2 and 3 on the role emotional, bodily pain, general health, vitality, social functioning, role emotional and physical component scores.

**Table 3:** Short form-12 mean scores comparing SIRS, uncomplicated sepsis and severe sepsis/septic shock.

Domains	Sterile SIRS	Uncomplicated sepsis	Severe sepsis/septic shock
Physical functioning	45.5 (9.9)	32.0 (7.7)*	25.6 (7.4)Δ
Role physical	43.9 (10.0)	38.3 (6.3)	29.5 (4.7)^Δ^ ‡
Bodily pain	46.3 (10.3)	36.9 (10.8)*	24.7 (4.4)^Δ^ ‡
General health	41.9 (11.3)	34.8 (8.8)	25.9 (4.1)^Δ^ ‡
Vitality	46.5 (8.6)	37.4 (8.2)*	34.0 (9.0)Δ
Social functioning	41.9 (13.3)	38.6 (10.7)	27.3 (5.5)^Δ^ ‡
Role emotional	37.7 (13.7)	41.9 (10.9)	30.3 (11.5)‡
Mental health	46.4 (12.5)	47.4 (15.6)	40.1 (11.7)
Physical componentscore	47 (10.3)	31.4 (7.4)*	20.9 (4.7)^Δ^ ‡
Mental component score	41.3 (13.8)	43.4 (12.4)	35.5 (10.2)

*denotes a significant difference between group 1 and 2. (p<0.05).

Δ denotes a significant difference between group 1 and 3. (p<0.05).

‡ denotes a significant difference between group 2 and 3. (p<0.05).

All results are presented as means (SD).

**Figure 2:**
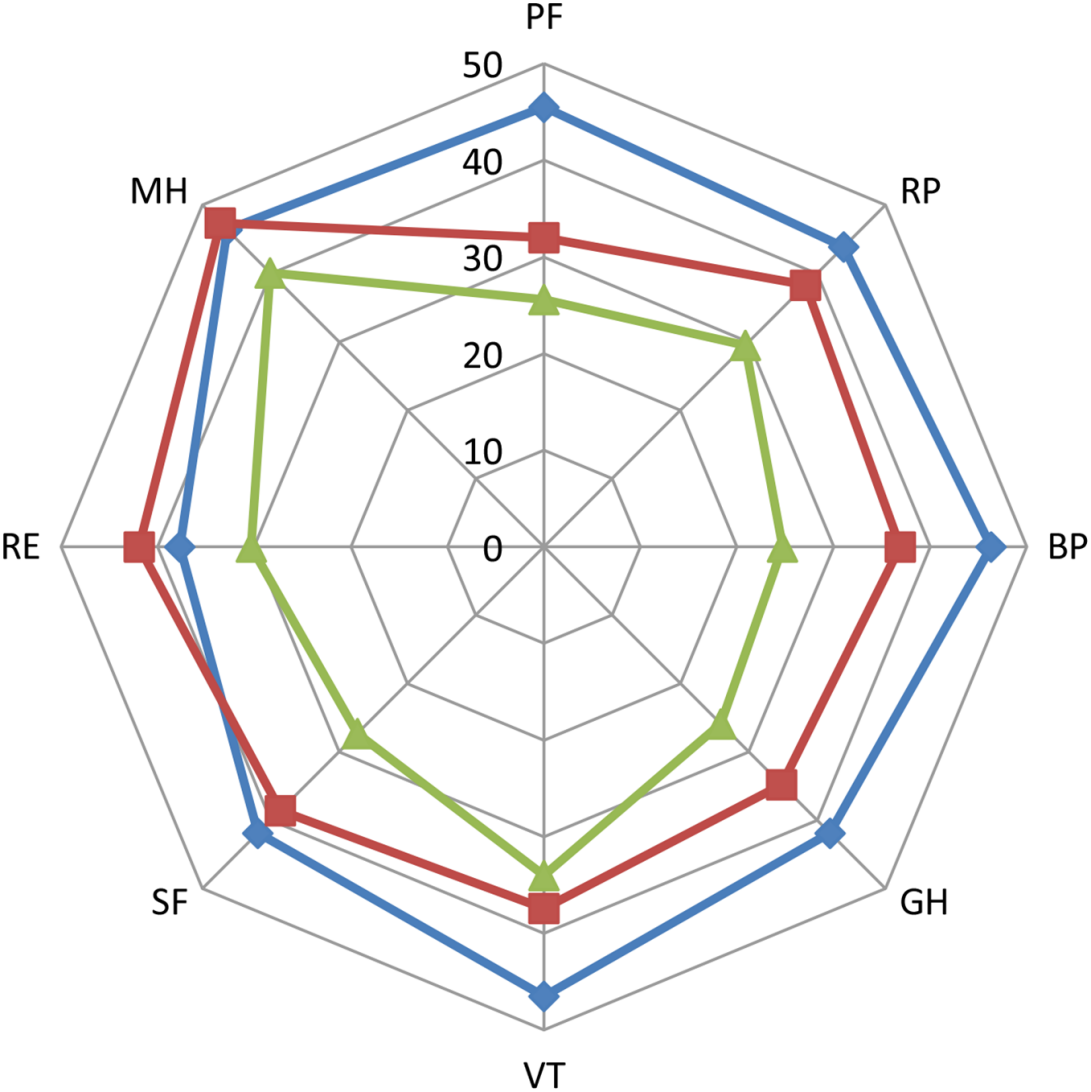
Health-Related Quality of Life in sterile SIRS, uncomplicated sepsis and severe sepsis/septic shock. Radar chart of the health-related quality of life in long-term survivors of Sterile SIRS (group 1– blue line with diamonds), uncomplicated sepsis (group 2– red line with squares) and severe sepsis/septic shock (group 3– green line with triangles). PF: physical functioning, RP: role limitation physical, BP: bodily pain, GH: general health, VT: vitality, SF: social functioning, RE: role limitation emotional, MH: mental health.

Fig. 2 is a radar chart that illustrates the difference in each of the eight health domains between the three different groups.

## Discussion

The results of our study have demonstrated that survivors of SIRS and sepsis continue to experience a reduced quality of life from between six months to two years post hospital discharge. The SIRS and sepsis patients reported a significantly lower quality of life than the Welsh population on all eight health domains in the SF-12v2 survey. There was no comparative data for the physical and mental component scores for the Welsh population data as this was generated using the earlier SF-36 version 1 survey which does not calculate these component scores. [15] The results of this study support previous research which has reported that quality of life is lower in survivors of sepsis [3]–[7].

Our results also reported significantly lower quality of life than the Mid/West Wales population and this is the first study to use such local geographical normative data for comparisons. Previous research has stated that it is imperative that accurate normative data is used in population studies in order to provide an indication of the health status of particular populations. [15] It is only using such specific normative data that the impact of clinical interventions can be accurately measured.

This is the first study investigating long-term quality of life in sepsis survivors that has categorised patients into SIRS, uncomplicated sepsis and severe sepsis/septic shock in order to reduce misclassification bias. The results of our study demonstrated that severe sepsis/septic shock patients have significantly lower physical health related quality of life than SIRS and uncomplicated sepsis patients. These results are significant when controlling for possible confounding variables age, pre-existing conditions, hospital length of stay and ICU length of stay. This supports previous research which found that sepsis survivors demonstrated similar decrements in quality of life long-term to other chronic diseases such as chronic obstructive pulmonary disease or congestive heart failure^3^ and also acute lung injury [5].

The mental component score did not demonstrate any significant difference between groups. Previous research has reported that mental component scores do not significantly differ between sepsis patients and the US normative data. [5], [17] Our results found that although there were no significant differences between the three groups, the mental component scores were still significantly lower compared to the Welsh population norms.

Knowledge of the reduced quality of life in sepsis survivors facilitates a more accurate targeting of patients for interventions such as ICU follow up clinics. The physical components of the survey in our respondents illustrated the most profound reductions in quality of life which suggests that rehabilitation programmes should be considered for these patients. Hofhuis et al (2008) reported that recovery of quality of life in ICU survivors starts as early as the time of ICU discharge and therefore rehabilitation programmes should start early [7].

There are a number of limitations of this study that should be considered. The main limitation is the lack of a baseline measurement of quality of life for the patients. It has been suggested that ICU patients’ pre-morbid quality of life has a large effect on quality of life after critical illness. [18] It could be argued therefore that the reduced quality of life reported in this study merely reflects a poor baseline, rather than being an effect of the sepsis. To overcome this limitation the patients could have been requested to complete a survey retrospectively. We believe that a retrospective assessment of pre-sepsis quality of life would have not improved this study however due to the issues of recall bias. [18] The results of this study found no difference in the pre-existing conditions between the three groups. This may suggest therefore that there was no difference in pre-morbid quality of life between the three groups and the differences reported long-term were actually due to sepsis.

Another limitation concerns the size of the groups as they did not quite reach the number stated in the sample size calculation which may have influenced the power of the study. A response rate of 80% is ideal for a quality of life study and that was not quite achieved in this study. [18] However analysis of non-responders and responders found no differences between the two groups. Furthermore, the follow up period in our study ranged from six months to two years and this may have influenced the study’s findings. There is no universal definition for ‘long-term’ in follow-up studies and time periods previously investigated range from three months [3] through to five years. [4] It has been recommended that a follow up period of one to two years will probably capture the most accurate data and it may be the limit for improvement in most quality of life dimensions [18].

In conclusion, our study is the first observational study to specifically focus on the different groups of SIRS and sepsis patients to assess long-term quality of life. Local population norms were used for comparison, rather than United Kingdom wide geographical norms that fail to reflect the intricacies of a country’s population. Quality of life was reported to be significantly reduced in SIRS, uncomplicated sepsis and severe sepsis/septic shock patients when compared to local normative data. As expected, more significant reductions in quality of life were found in severe sepsis/septic shock patients than in uncomplicated sepsis and SIRS patients, when controlling for age, pre-existing conditions, hospital and ICU length of stay. Despite the limitations, this study encourages further multi-centred investigation into the long-term quality of life in survivors of SIRS and sepsis.
